# Evaluation of the DAMSUN-HF trial: the role of an artificial intelligence stethoscope in detecting reduced ejection fraction in patients living in a low-resource region

**DOI:** 10.1007/s10741-025-10591-2

**Published:** 2026-01-05

**Authors:** Dmitry Abramov, Baljash S. Cheema, Kalliopi Keramida, Kaveh Hosseini, Marat Fudim, Abdul Mannan Khan Minhas

**Affiliations:** 1 Division of Cardiology, Loma Linda University Health, Loma Linda, CA USA; 2 Center for Artificial Intelligence, Northwestern Medicine, Bluhm Cardiovascular Institute, Chicago, IL USA; 3 Feinberg School of Medicine, Northwestern University, Chicago, IL USA; 4 Cardiology Department, General Anti-Cancer Oncological Hospital Agios Savvas, Athens, Greece; 5 Department of Cardiology, Athens University Hospital Attikon, National and Kapodistrian University of Athens Medical School, Athens, Greece; 6 Department of Cardiology, Copenhagen University Hospital - Herlev and Gentofte, Copenhagen, Denmark; 7 Center for Translational Cardiology and Pragmatic Randomized Trials, Department of Biomedical Sciences, Faculty of Health and Medical Sciences, University of Copenhagen, Copenhagen, Denmark; 8 Department of Medicine, Duke University Medical Center, Durham, NC USA; 9 Duke Clinical Research Institute, Durham, NC USA; 10 Section of Cardiology, Department of Medicine, Baylor College of Medicine, Houston, TX USA

**Keywords:** Ejection fraction, Artificial intelligence, Heart failure, Echocardiography

## Abstract

Evaluation of ejection fraction (EF) is paramount for patients with symptoms of heart failure. While transthoracic echocardiography (TTE) is the most common way to evaluate EF, recent advances in artificial intelligence (AI) have opened the door for alternative methods to screen for reduced EF with smaller and more portable technology. The DAMSUN-HF study evaluated the accuracy of an AI-based stethoscope for detecting reduced EF (≤40%) in patients with symptoms of heart failure in a region with geographic and economic barriers to obtaining timely TTE. This mini-review examines the DAMSUN-HF study and highlights the potential clinical implications of the study findings.

Heart failure (HF) affects more than 50 million people worldwide and remains one of the leading causes of morbidity and mortality across both developed and developing countries[1]. Heart failure diagnosis relies on a thorough history and physical exam, laboratory values, and cardiac imaging techniques. Transthoracic echocardiography (TTE) is the cornerstone for the diagnosis of HF as it allows for both the assessment of ejection fraction (EF) as well as for identification of potential structural causes. Although TTE is widely available in most developed countries, regional disparities in developing countries and potentially in rural regions in developed countries may result in reduced access or financial burdens to obtaining a TTE. Consequently, novel technologies based on artificial intelligence (AI) have been developed to bridge the gap in TTE availability, which may improve the identification and subsequent management of patients with suspected HF[2]. One promising technology relies on AI algorithms using data from an electronic stethoscope, which is able to simultaneously record a short single-lead electrocardiogram and heart sounds, which are then processed by a deep convolutional neural network trained on large datasets of paired recordings and echocardiograms[3]. The derivation was supervised using echo-derived left ventricular EF binarized at a threshold of ≤40% vs >40%, to learn latent waveform and audio features associated with reduced systolic function. For a new recording, the algorithm outputs the probability that LVEF is ≤40% and classifies the patient as having “low EF” or “normal EF,” thereby functioning as a binary screen for reduced EF rather than providing a continuous EF estimate. Building algorithms with an EF cutoff of ≤40% is clinically meaningful as EF ≤40% is typically used to define systolic HF which may be amenable to treatment through multiple classes of guideline-directed medical therapies. AI stethoscope technology from Eko Health (Oakland, CA) was previously tested in the United States among patients presenting for a TTE and demonstrated sensitivity and specificity around 80% for identification of patients with EF ≤40%[3], with the Eko Health SENSORA^TM^ AI stethoscope platform gaining Food and Drug Administration clearance for detection of low EF. However, stethoscope AI-based algorithms for detection of low EF have not yet been tested in settings where they are potentially most useful, such as in developing nations with limited access to other imaging technologies.

To fill this knowledge gap, the Detection and Management of Heart Failure with SENSORA^TM^in Underserved Nations–Heart Failure Study (DAMSUN-HF) sought to prospectively evaluate the accuracy of an AI assisted electronic stethoscope in patients with symptoms potentially indicative of HF to classify EF into categories of ≤ or> than 40%[4]. The study evaluated 115 adults, age ≥18 years, in the African country of Ghana who presented with dyspnea, orthopnea, or edema. The mean age was 62 ± 16 years and 53% were women. The cohort had a high burden of associated comorbidities including hypertension (68%), diabetes (20%), and known HF (38%). The study cohort was highly symptomatic with many experiencing New York Heart Association Class III (44%) or class IV (28%) symptoms. Utilization of common HF therapies including beta-blockers and angiotensin system inhibitors was high at 72% and 69%, respectively. All patients were initially evaluated with the AI stethoscope and subsequently referred to a tertiary center where a TTE was performed and interpreted in a blinded fashion by cardiologists.

The AI stethoscope evaluation was completed in 100% of participants and 95% underwent a subsequent TTE within 7 days (median 5 days). Overall, 64 participants (59%) had an EF ≤40%. The AI algorithm currently identified 62 out of 64 patients with EF ≤40%, leading to a sensitivity of 97%, a specificity of 76%, a positive predictive value of 85%, a negative predictive value of 94%, and a receiver-operating curve AUC of 0.92 (95% confidence interval of 0.87-0.97). Diagnostic performance was consistent across subgroups based on sex and age. The authors concluded that implementation of the AI assisted stethoscope was feasible in an underserved setting and achieved high diagnostic accuracy.

The DAMSUN-HF study highlights the evolving field of AI-assisted cardiac diagnostics and is an important advancement for attempts to implement AI technologies in regions with limited access to tertiary cardiac care. Particularly, implementation of AI assisted identification of reduced EF can be performed in rural or other low-resource settings and by clinicians with a broad range of clinical training and diagnostic expertise. AI results indicating a high likelihood for low EF may consequently improve risk stratification and facilitate higher-yield referrals for additional cardiac testing, which may better identify patients who may derive benefit from treatments.

The results of this study need to be interpreted in the context of important limitations (Fig. 1). Operator training and learning-curve effects were not described, even though similar AI stethoscope algorithms are explicitly designed for use by non-specialist clinicians in primary care and task-shifted models[5]. Assessment of EF by TTE has high reader variability, including around the 40% threshold[6], and presenting results using an absolute cutoff may affect conclusions about the accuracy of the algorithm. Reader variability for EF was not reported in DAMSUM-HF. The TTE findings of the 11 patients with false positive findings were not presented, although prior data from the Eko AI-assisted stethoscope identified several main reasons for reduced specificity including true EF being mildly above the cutoff range, the presence of atrial fibrillation, or the presence of conduction system abnormalities[3]. DAMSUN-HF included a small cohort of highly symptomatic individuals who may derive significant benefit from TTE irrespective of EF to identify other causes of HF symptoms including valvular heart disease (with the prevalence of valve disease not reported in this study). In fact, AI detection tools that focus on identifying underlying causes of HF, such as valve disease, arrhythmias, or particular causes of infiltrative cardiomyopathy may be additive to assessment of EF and potentially even more valuable for clinical decision making[7–9]. Consequently, it may be detrimental to interpret the high sensitivity and high negative predictive value reported in this study in the clinical context as reassurance about EF and potentially miss other important and treatable causes of HF symptoms. Moreover, 38% of participants in DAMSUN-HF had pre-existing HF – a proportion that raises questions about the incremental diagnostic value of AI auscultation and suggests that this cohort is more representative of triage rather than screening. While the high sensitivity and negative predictive value support its use as a triage tool, broader external validation - especially in asymptomatic populations, in individuals with vague symptoms, and in those with multimorbidity - is essential before widespread implementation. The performance of these tools in obese patients, patients with chronic obstructive pulmonary disease or prominent pulmonary findings, concomitant valvular disease and under varying hemodynamic conditions such as uncontrolled hypertension or hypotension, remains largely unexplored and may influence acoustic signals and diagnostic accuracy. The role of combining AI-assisted stethoscopes with other AI technology for identification of EF and HF, such as electrocardiogram-based or cost-effective ultrasound-based algorithms, will also require further evaluation. Finally, implementation at scale must consider the cost of devices and software subscriptions, which currently remain several hundred euros per unit—an important barrier for widespread use in low-income regions.

**Fig. 1:**
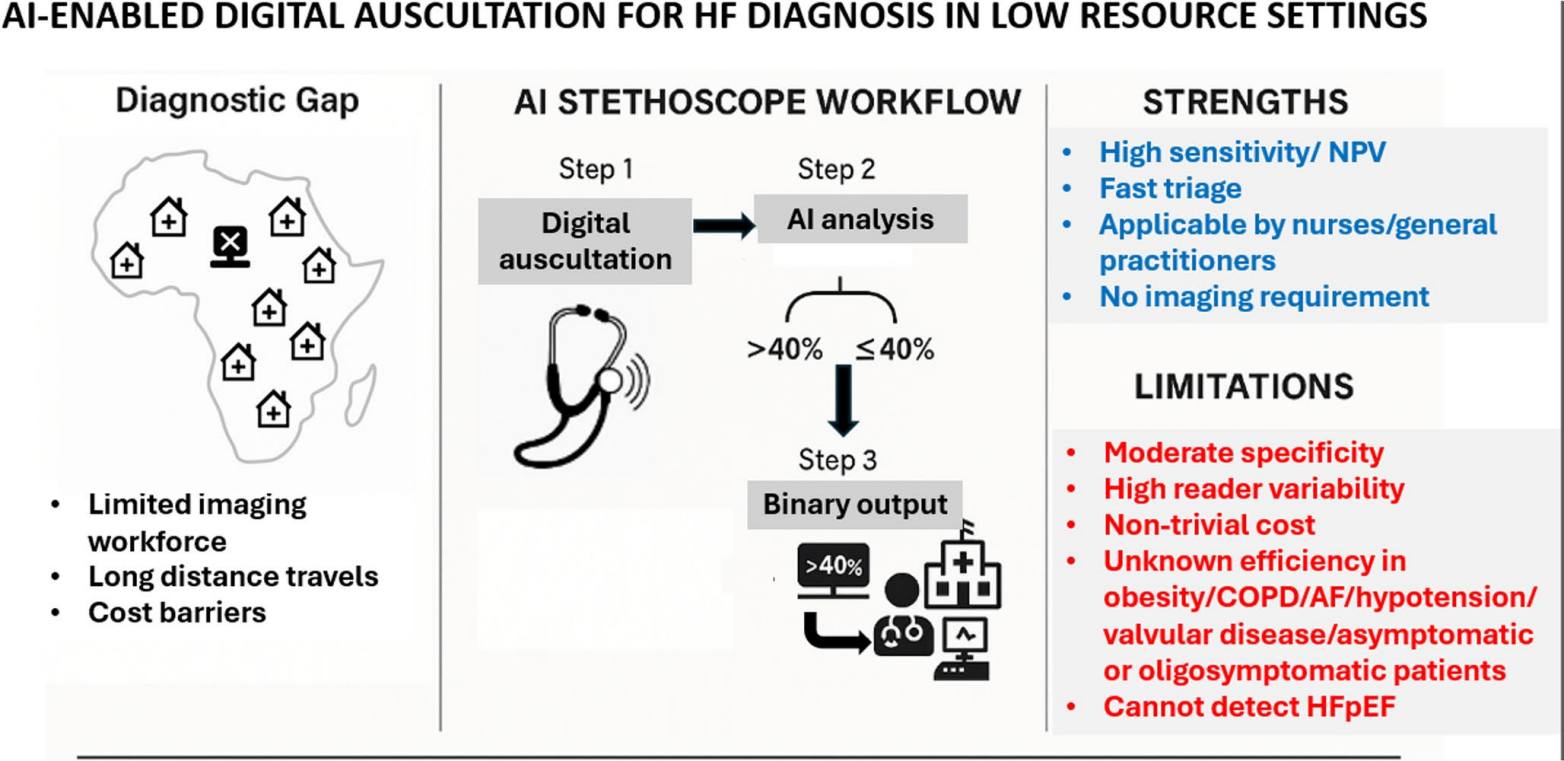
DAMSUN HF Trial: Strenghts and Limitations

In conclusion, studies like DAMSUN-HF provide important real-world experience on currently available AI technology for HF diagnosis, in low resource regions where access to imaging is limited. As AI-assisted algorithms only continue to improve, a focus on technology costs, ease of use, and diagnostic accuracy will be important as these algorithms strive to enter clinical care alongside practicing clinicians.

## Data Availability

No datasets were generated or analysed during the current study.
